# Tumour necrosis factor alpha promotes secretion of 14-3-3η by inducing necroptosis in macrophages

**DOI:** 10.1186/s13075-020-2110-9

**Published:** 2020-02-12

**Authors:** Gulzhan Trimova, Kaoru Yamagata, Shigeru Iwata, Shintaro Hirata, Tong Zhang, Fumi Uemura, Minoru Satoh, Norma Biln, Shingo Nakayamada, Walter P. Maksymowych, Yoshiya Tanaka

**Affiliations:** 1 The First Department of Internal Medicine, School of Medicine, University of Occupational and Environmental Health, Japan,1-1 Iseigaoka, Yahata-nishi-ku, Kitakyushu, Fukuoka 807-8555 Japan; 2 Department of Clinical Immunology and Rheumatology, Hiroshima University Hospital, Hiroshima, Japan; 3 Department of Clinical Nursing, School of Health Sciences, University of Occupational and Environmental Health, Kitakyushu, Japan; 4 Augurex Life Sciences Corp. Executive, North Vancouver, BC Canada; 5 Department of Medicine, University of Alberta, Edmonton, AB Canada

**Keywords:** Rheumatoid arthritis, 14-3-3η, TNF-α, Necroptosis, Macrophage

## Abstract

**Background:**

14-3-3η is an intracellular protein also detected in the serum and synovial fluid of patients with rheumatoid arthritis (RA). It is closely related to disease activity and anti-cyclic citrullinated peptide antibody levels. However, the main source of 14-3-3η and the mechanism of its release into the extracellular space remain unclear. Addressing these two points was the main goal of the current study.

**Methods:**

The source of 14-3-3η was investigated by immunostaining RA synovial tissue. Fibroblast-like synoviocytes, CD4^+^ cells, and macrophages were selected as candidates among the various cell types in the synovial tissue. Phosphorylation of mixed-lineage kinase domain-like pseudokinase (MLKL) and cell death of macrophages were studied by phalloidin staining and electron microscopy after stimulation with an oxidative stress inducer (diamide) or tumour necrosis factor (TNF)-α. Extracellular 14-3-3η protein levels were examined by western blotting.

**Results:**

Macrophages from the synovial tissue from RA, but not osteoarthritis, showed dense and widespread cytoplasmic staining for the 14-3-3η protein, co-localized with peptidylarginine deiminase 4. Swelling and membrane rupture of macrophages were induced by treatment with TNF-α, but not interleukin (IL) 6/soluble IL-6 receptor (sIL-6R). Increased MLKL phosphorylation followed by necroptosis was also induced in TNF-α-stimulated macrophages. Necrostatin-1, a necroptosis inhibitor, antagonized MLKL phosphorylation. High levels of 14-3-3η were detected in the culture supernatants of macrophages stimulated with diamide and TNF-α, but not IL-6/sIL-6R.

**Conclusions:**

Macrophages that highly express 14-3-3η undergo TNF-α-induced necroptosis with damage to the cellular structure, resulting in the secretion of 14-3-3η into the extracellular space. The current study provides a novel mechanism for 14-3-3η level increase in the RA synovial fluid.

## Background

Rheumatoid arthritis (RA) is a systemic autoimmune disease characterized by chronic inflammation of the synovium and joint destruction [1–5]. Initially, the levels of inflammatory cytokines, such as tumour necrosis factor (TNF)-α and interleukin (IL) 6, and proteases, such as matrix metalloproteinase (MMP) 3, are increased in the synovial fluid of patients with RA. Lymphocyte infiltration of the synovial tissue plays an important role in the pathogenesis of the disease [6, 7]. Therefore, TNF inhibitors, IL-6 receptor (IL-6R) inhibitors, cytotoxic T lymphocyte-associated protein 4, and low-molecular compound Janus kinase (JAK) inhibitors have been developed and used clinically [8–12]. However, not all patients with RA achieve remission, and some may relapse. To address this problem, it is important to find new molecules that play a crucial role in the pathogenesis of RA and could constitute new therapeutic targets.

Chaperone 14-3-3η molecules, which are usually located intracellularly, are unexpectedly detected in the synovial fluid of RA patients and thus attracted scientific attention [13]. 14-3-3η molecules have been extracted from the brain of cattle and are detected in many other species [14]. 14-3-3 proteins are generally localized in the nucleus, cytoplasm, and cell membrane [15]. Seven isoforms of these proteins have been identified (β, ε, η, γ, τ, σ, and ζ), encoded by various genes in mammals. Further, the 14-3-3 protein binds to target proteins and regulates their modification, cellular localization, and enzymatic activity. In many cases, 14-3-3 appears to interact with a specific phosphorylated threonine-serine motif [15]. 14-3-3 proteins are involved in diverse biological events, such as cell cycle progression, intracellular protein trafficking, apoptosis, DNA damage repair, DNA replication, and transcriptional regulation [16]. It has been reported that siRNA-mediated suppression of 14-3-3 expression in a glioma cell line results in the suppression of cell proliferation, followed by apoptosis [17]. These observations indicate that the 14-3-3 protein plays an important role in the regulation of various intracellular events.

The importance of 14-3-3η in pathological processes relevant to RA was first proposed when 14-3-3η was detected in the serum of patients with early RA. Subsequently, the protein was found to be an early diagnostic marker of RA, showing complementarity with anti-citrullinated protein antibodies (ACPA) and rheumatoid factor [13, 18–20]. We have reported that the serum levels of 14-3-3η correlate with disease activity markers (DAS28-ESR, CDAI, and SDAI scores) in patients with RA and also with the serum levels of ACPA. Adalimumab (ADA), an anti-TNF antibody, but not tocilizumab (TCZ), an anti-IL-6R antibody, reduces serum levels of 14-3-3η and ACPA [21]. Since ACPA is an autoantibody that targets proteins citrullinated by peptidylarginine deiminase 4 (PAD4), PAD4 may induce 14-3-3η citrullination. Furthermore, when the human monocytic cell line THP1 is stimulated with recombinant 14-3-3η, the expression of genes such as ones encoding TNF-α, IL-6, MMP-3, and RANKL is induced [22]. In addition, 14-3-3η appears to be a mediator of the RA pathogenesis as well as a diagnostic biomarker.

Of note, 14-3-3η is detected in the central nervous system and synovial tissue [18, 23]. However, the cell types that are the primary source of 14-3-3η in patients with RA and the mechanism of 14-3-3η secretion into the joint fluid remain unknown. 14-3-3η may occur in the RA synovial fluid as a result of exocytosis or cell death. In RA, apoptosis of inflammatory cells is reduced, and the expression of necrosis markers in a collagen-induced mouse model of arthritis is increased in cells proximal to the joints [24]. The pro-inflammatory cytokine TNF-α, which is considered a key cytokine for the pathogenesis of RA, causes death of various cell types. In particular, its involvement in necroptosis attracts much attention [25–27]. Therefore, we hypothesized that necroptosis may be involved in the secretion of 14-3-3η. In the current study, we aimed to define the source of the 14-3-3η protein by immunostaining the synovial tissue and to define the mechanism of cell death of macrophages and 14-3-3η secretion in patients with RA.

## Materials and methods

### Cell culture

Monocytes were purified from Ficoll-isolated peripheral blood mononuclear cells and cultured in 6-well plates at a density of 100,000 cells/cm^2^ in RPMI-1640 medium (Wako, Wakayama, Japan). They were differentiated into macrophages by treating with 100 ng/ml macrophage colony-stimulating factor (Peprotech, Rocky Hill, NJ). Macrophages were cultured in RPMI-1640 medium containing 10% (v/v) foetal bovine serum (Tissue Culture Biological, Tulare, CA) and antibiotics (1% (v/v) penicillin and streptomycin, Life Technologies, Carlsbad, CA), seeded at a density of 100,000 cells/cm^2^ in 6-well plates and incubated at 37 °C under 5% CO_2_ atmosphere, as described previously [28].

### Antibodies and reagents

The following antibodies were purchased and used for immunohistochemistry (IHC), immunofluorescence (IF), immunocytochemistry (ICC), and immunoblotting (IB) experiments: mouse monoclonal antibodies against 14-3-3η (1:200; AAM-11-0003, Augurex, Vancouver, BC, Canada) and 14-3-3γ (1:200; sc-731, Santa Cruz Biotechnology, Dallas, TX) for IHC, CD4 (1:200; ab67001, Abcam, Cambridge, MA) for IF, CD55 (1:200; ab1422, Abcam) for IF, 14-3-3η (1:200; NBP1-92691SS, Novus Biologicals, Centennial, CO) and phospho-Akt (1:200; Ser473) (#9271 Cell Signaling Technology, Danvers, MA) for ICC, and β-actin (1:10,000; A1978, Sigma-Aldrich, St. Louis, MO) for IB; rabbit monoclonal antibodies against CD68 (1:200; 11192-RP02, Sino Biological Inc., Wayne, PA), PAD4 (1:200; 17373-1, ProteinTech, Rosemont, IL), and phospho-RIP3 (Ser227) (1:200; 93654S, CST) for IF and ICC, and MLKL (1:2000; phospho S358, ab187091, Abcam) for IB; rabbit polyclonal antibodies against MLKL (1:2000; ab194699, Abcam) for IB.

Rhodamine-labelled anti-mouse IgG antibody (F31663, Thermo Scientific, Suwanee, GA), FITC anti-rabbit IgG antibody (F9887, Sigma-Aldrich, St. Louis, MO), 4′,6-diamidino-2-phenylindole (DAPI; sc-3598, SCB), and rhodamine phalloidin (R415, Thermo Fisher Scientific, Waltham, MA) were used for IF. Anti-mouse IgG and anti-rabbit IgG secondary antibodies conjugated with horseradish peroxidase (HRP) polymer for IB were obtained from GE Healthcare (Little Chalfont, UK).

TNF-α, IL-6, soluble IL-6 receptor (sIL-6R), and IL-21 were purchased from PeproTech, Miltenyi Biotech (Auburn, CA), R&D Systems (Minneapolis, MN), and PeproTech, respectively. Recombinant 14-3-3η, diamide, actinomycin D, and lipopolysaccharide (LPS) were from Abcam, Sigma-Aldrich, and Wako, respectively. Tofacitinib (TOF) and etanercept (ETN) were kindly provided by Pfizer (New York, NY); ADA was provided by Abbvie (Tokyo, Japan). Necrostatin-1 (Nec-1) or zVAD-FMK was purchased from Abcam (ab141053) or Selleckchem (S7023) (Houston, TX).

### Histological examination

The synovial tissue of patients (ethical approval was obtained from the University of Occupational and Environmental Health, Japan Ethics Committee) with RA and osteoarthritis (OA) was obtained during operation, fixed in 10% formaldehyde, and embedded in paraffin. Samples were subjected to histological examinations, including IHC and IF.

IHC was performed as described previously, with slight modifications [29]. Briefly, antigen retrieval was performed by soaking tissue specimens from surgery on slides in 5 mM sodium citrate solution in phosphate-buffered saline (PBS) containing 0.05% (v/v) Tween 20 (PBST) (pH 6.0). Slides were blocked with serum-free protein block (Dako, 2016-08) for 60 min at 25 °C and then incubated for 2 h with mouse monoclonal antibodies specific for 14-3-3η and 14-3-3γ, which were diluted 1:200 in Can Get Signal Immunostain Solution A (NKB-501; Toyobo, Osaka, Japan). The slides were washed three times with PBST, incubated with anti-mouse IgG secondary antibodies conjugated with HRP polymer (DakoCytomation, Glostrup, Denmark) for 1 h, and then visualized by treatment with the DAB chromogen (3,3′ diaminobenzidine, DakoCytomation, #K3465) for 8 min, according to the manufacturer’s instructions. The nucleus was visualized using Mayer’s haematoxylin (1:1000 dilution in PBST; Merck, Darmstadt, Germany). For mounting, the sections were rinsed in water, dehydrated in graded ethanol series (90% ethanol for 30 s × 3 and 100% ethanol for 30 s × 3), cleared in xylene (for 30 s × 2), and sealed using Multi mount 480 (FM48001; Matsunami, Kishiwada, Japan).

IF was performed as described previously [30]. Biopsy specimens were incubated with the respective antibodies specific against 14-3-3η, PAD4, CD4, CD55, and CD68. The nucleus was stained with DAPI (5 μg/ml).

### Immunocytochemistry

ICC was performed as described previously, with slight modifications [28]. Primary macrophages were cultured on coverslips. The cells were pre-treated for 24 h with nec-1 or TOF. They were then treated, or not, with TNF-α, diamide, IL-6/sIL-6R, or LPS, as indicated, and fixed with 1% paraformaldehyde in PBS (163-20145, Wako) for 15 min at room temperature. The cells were then rinsed once with PBS, and permeabilized with PBS containing 0.1% (v/v) Triton X-100 for 15 min at room temperature. After blocking with PBS containing 10% (v/v) foetal bovine serum for 30 min at room temperature, the cells were incubated with antibodies specific against 14-3-3η (1:200), PAD4 (1:200), pRIP3 (1:200), pAkt (1:200), or phalloidin (1:140) diluted with PBS, for 2 h at 25 °C. The cells were rinsed three times with PBS, then incubated with the secondary antibodies, rhodamine goat anti-mouse (1:500) and/or FITC goat anti-rabbit IgG (1:500), and DAPI (5 μg/ml) diluted with PBS for 1 h at room temperature. They were then rinsed three times with PBS and mounted using ProLong Diamond Antifade Mountant (Invitrogen, Suwanee, GA). The experiments were independently repeated three times. Samples were visualized using an all-in-one fluorescence microscope (BZ-X700; Keyence, Itasca, IL).

### Transmission electron microscopy

The experiment was performed as described previously [31]. Macrophages were fixed with PBS containing 2% glutaraldehyde and 2% paraformaldehyde, followed by incubation in 1% OsO_4_, and dehydrated in a graded alcohol series. After freeze-drying in *t*-butyl-alcohol, the samples were examined under a transmission electron microscope (TEM) (TM3000; Hitachi, Tokyo, Japan).

### Western blotting

Whole-cell lysates (WCLs) prepared from primary macrophages were resolved by SDS-PAGE and then immunoblotting (IB), as described previously [29]. Briefly, the cells were solubilized in lysis buffer [50 mM Tris-HCl (pH 7.4), 0.5% (v/v) Nonidet P-40, 150 mM NaCl, 5 mM EDTA, 50 mM NaF, 1 mM Na3VO4, 1 mM phenylmethylsulfonyl fluoride, 10 mg/ml leupeptin, and 10 mg/ml aprotinin] and centrifuged at 12,000×*g* for 20 min at 4 °C. The supernatant obtained after the centrifugation was used as WCL. WCL proteins were separated by SDS-PAGE on 10% or 13% Tris-glycine gel and transferred onto nitrocellulose (AmershamProtran 0.2 μm NC; GE Healthcare, Chicago, IL). The membranes were immunoblotted using mouse monoclonal antibodies specific against phosphorylated (p) MLKL or MLKL. Bound antibodies were visualized using anti-rabbit IgG secondary antibodies conjugated with HRP polymer (GE Healthcare, Chicago, IL) using chemiluminescence reagents (ECL™ prime western blotting detection reagent; GE Healthcare, Chicago, IL).

Proteins in the gel were stained with Coomassie Brilliant Blue (CBB) R-350 (GE Healthcare, Chicago, IL) according to the manufacturer’s instruction.

WB protein bands were quantified by using ImageJ software as described previously [32].

### Detection of extracellular 14-3-3η

To examine 14-3-3η secretion by macrophages, macrophages were pre-treated for 30 min with or without nec-1, z-VAD-FMK (pan-caspase inhibitor), or TOF, and then stimulated with TNF-α, diamide, LPS, IL-6/sIL-6R, IL-1β, or IL-21 in 6-well plates for 24 h. Culture supernatants were concentrated using Microcon (MRCPRT010; Millipore, Darmstadt, Germany) according to the manufacturer’s instructions. 14-3-3η levels in culture supernatants were determined by WB, as described above.

### Statistical analysis

All quantitative data are expressed as the mean ± standard deviation (SD). Differences between the two groups were tested for statistical significance by Student’s unpaired two-tailed *t* test; *P* value < 0.05 was considered significant. Statistical analyses were performed using SPSS statistical software (version 21.0; SPSS, Inc., Chicago, IL).

## Results

**Fig. 1:**
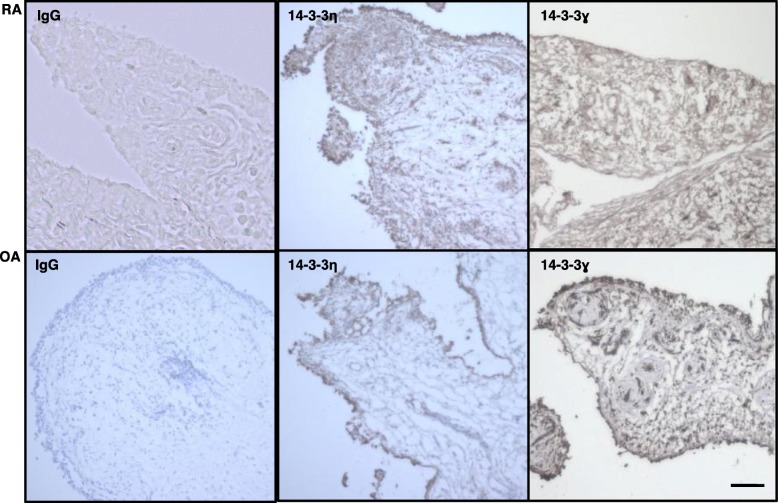
Higher expression of 14-3-3η^+^ and fewer 14-3-3ɣ^+^ cells were detectable in the RA synovium than in OA synovium. Synovium specimens of patients with RA (*n* = 3) or OA (*n* = 3) were incubated with the indicated primary antibodies and then visualized using DAB chromogen. Representative images from three independent experiments are shown. Panels labelled IgG, isotype control. Scale bar, 50 μm

The synovial tissue specimens from RA and OA individuals were analysed by IHC using an antibody against 14-3-3η (Fig. 1). Patient information is provided in Additional file 1: Table S1. 14-3-3η was detected in the surface layer as well as inside the RA synovial tissue. By contrast, it was detected only in the surface layer in OA synovial tissue. Further, 14-3-3γ was only weakly detected in the RA synovial tissue, although it was detected in both the surface layer and inside the OA synovial tissue. The IgG isotype control did not react with the synovial tissue.

**Fig. 2:**
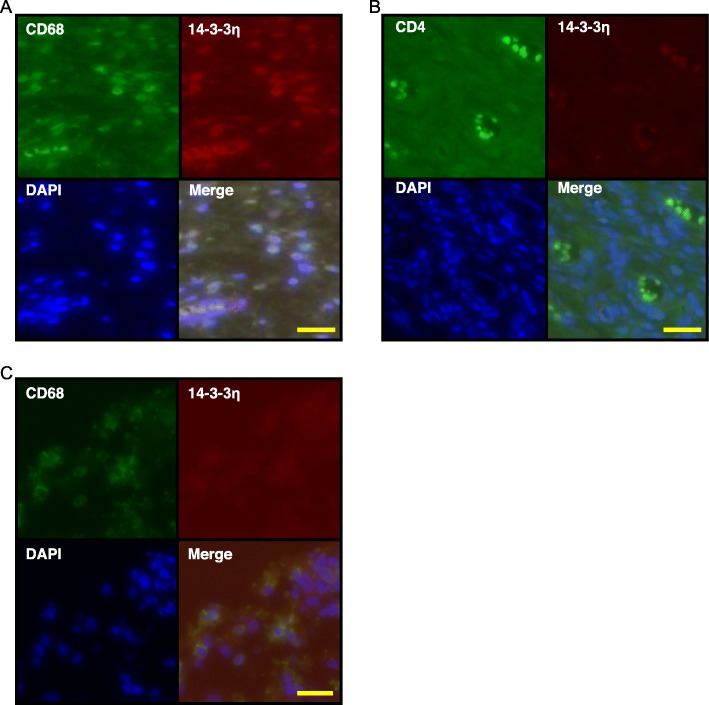
14-3-3η is expressed in CD68^+^ cells but not in CD4^+^ T cells from the synovium of RA patients. Synovium specimens of patients with RA (*n* = 3) were analysed by IF using specific antibodies against 14-3-3η and CD68 (**a**) or CD4 (**b**). **c** Lung tissue specimens of patients with RA (*n* = 3) were analysed using specific antibodies against 14-3-3η and CD68. Representative images of three independent experiments are shown. Scale bar, 50 μm

To identify the source of 14-3-3η in the RA synovial tissue, double-fluorescent immunostaining was performed using antibodies against various cell markers and 14-3-3η (Fig. 2). Macrophages staining positive for CD68 (Fig. 2a), but not CD4^+^ T cells (Fig. 2b), also stained positive for 14-3-3η. Furthermore, 14-3-3η was not detected in CD68^+^ cells in the RA lung tissue specimens (Fig. 2c). These observations indicate that the 14-3-3η protein was expressed in CD68^+^ cells in the RA synovial tissue, showing organ specificity.

**Fig. 3:**
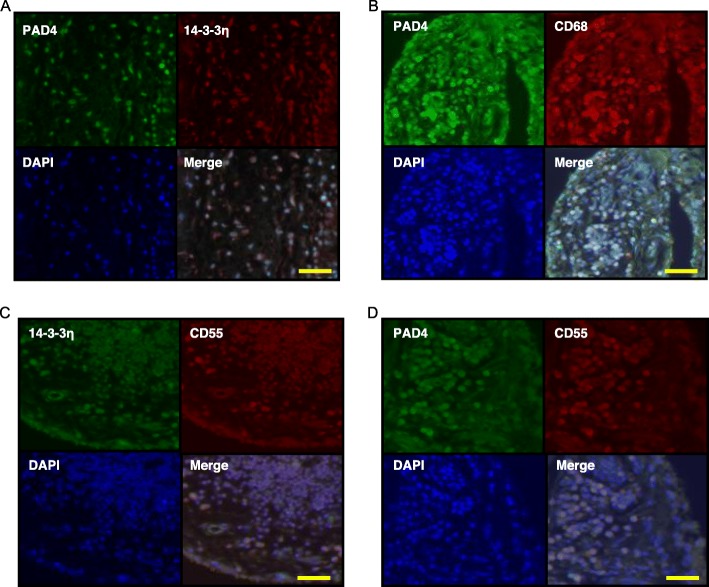
PAD4 is localized in CD68^+^ cells and CD55^+^ cells in the RA synovium. Synovium (*n* = 3) specimens of patients with RA were assessed by IF using primary antibodies against PAD4 and 14-3-3η (**a**), PAD4 and CD68 (**b**), 14-3-3η and CD55 (**c**), or PAD4 and CD55 (**d**) and by FITC or rhodamine-labelled secondary antibodies. Representative images from three independent experiments are shown. Scale bar, 50 μm

In the peripheral blood of patients with RA, the 14-3-3η levels are associated with ACPA levels [20, 21, 26, 27]. To examine the localization of PAD4 and 14-3-3η, the RA synovial tissue was IF-stained using an antibody against PAD4 (Fig. 3). 14-3-3η was detected in many PAD4^+^ cells (Fig. 3a); CD68 was also detected in PAD4^+^ cells (Fig. 3b). In addition, cells positive for CD55, a marker for fibroblast-like synoviocytes, expressed both 14-3-3η protein (Fig. 3c) and PAD4 (Fig. 3d). This indicated that PAD4 and copious amounts of 14-3-3η were expressed in macrophages and fibroblast-like synoviocytes in the synovial tissue of patients with RA.

**Fig. 4:**
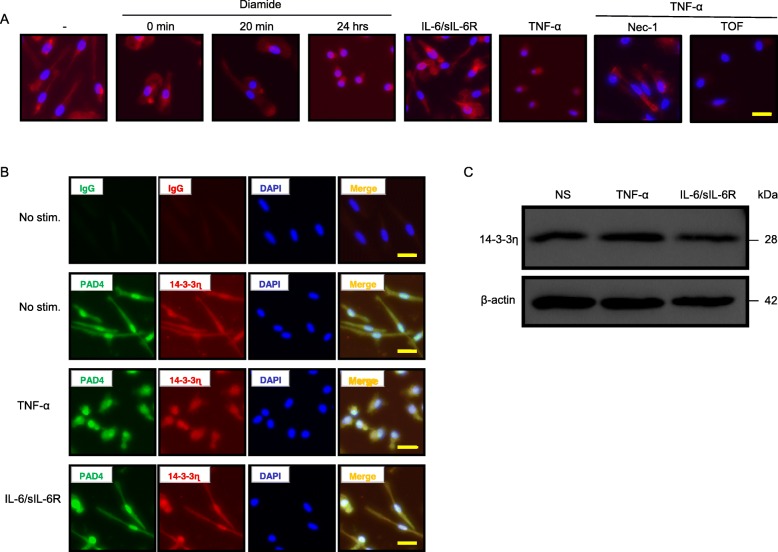
TNF-α induces abnormal cell morphology of macrophages from HD. Macrophages were cultured with or without diamide (100 nM; for the indicated time; *n* = 3), IL-6/sIL-6R (10 ng/ml; 24 h; *n* = 3), and TNF-α (10 ng/ml; 24 h; *n* = 3) in the presence or absence of nec-1 (20 nM; *n* = 3) or TOF (300 nM; *n* = 3). The cells were then stained with specific antibodies against phalloidin (**a**) or isotype control, PAD4, or 14-3-3η (**b**), and DAPI (**a**, **b**). **c** 14-3-3η levels were detected by WB. Representative images from three independent experiments are shown. Scale bar, 50 μm

Macrophages derived from peripheral blood mononuclear cells of healthy donors (HD) were next treated with an oxidative stress inducer diamide. The cytoskeletal protein phalloidin was then detected using a fluorescent-labelled antibody (Fig. 4a). Diffuse staining was observed at 0 min and 20 min; by contrast, after 24-h treatment, the staining was only apparent surrounding the nucleus. When the cells were stimulated with IL-6/sIL-6R, phalloidin was detected in the cytoplasm; however, it was detected only surrounding the nucleus after TNF-α stimulation. Pre-treatment of macrophages with nec-1, a necroptosis inhibitor, blocked the effect of TNF-α. However, the non-selective JAK inhibitor TOF did not antagonize necroptosis of macrophages, indicating that the JAK-STAT pathway was not secondarily involved in necroptosis induced by TNF-α. Next, macrophages were stimulated with TNF-α or IL-6/sIL-6R for 24 h and probed by double-fluorescent immunostaining to detect PAD4 and 14-3-3η (Fig. 4b). In unstimulated macrophages from HD, PAD4 and 14-3-3η were clearly detected in both the nucleus and cytoplasm, and the isotype control IgG did not stain the cells. Stimulation of macrophages with IL-6/sIL-6R resulted in cells with similar staining features as those of cells without stimulation. By contrast, PAD4 and 14-3-3η were detected mainly in the nucleus of macrophages stimulated with TNF-α. This suggested a role of TNF-α in the cellular distribution of PAD4 and 14-3-3η. The effect was not associated with the upregulation of 14-3-3η protein levels, as TNF-α, IL-6/sIL-6R, IL-1β, and IL-21 did not affect the expression of endogenous 14-3-3η (Fig. 4c and Additional file 2: Figure S1).

**Fig. 5:**
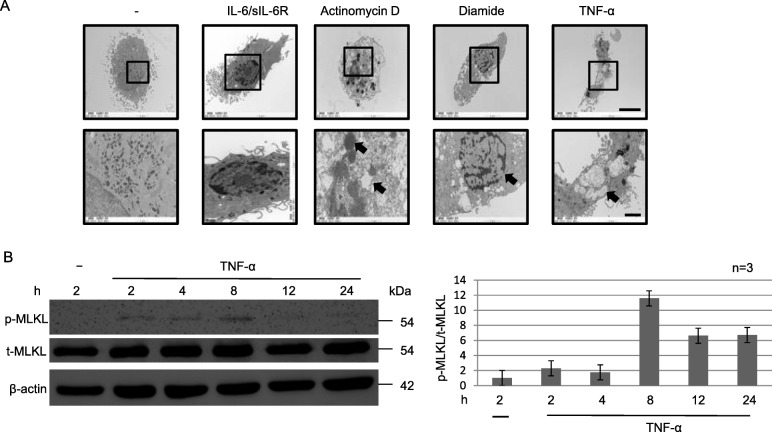
TNF-α induces cell death of macrophages from HD. **a** Macrophages were cultured with or without IL-6/sIL-6R (10 ng/ml; *n* = 3), actinomycin D (1 μg/ml; *n* = 3), diamide (1 mM, *n* = 3), or TNF-α (100 ng/ml; *n* = 3) for 24 h and then analysed by TEM. **b** WCLs prepared from macrophages cultured with or without TNF-α (100 ng/ml; *n* = 3) were analysed by IB using specific antibodies against the total or phosphorylation form of MLKL or β-actin. Quantification data for representative images from three independent experiments (*n* = 3) are shown. Scale bar, 5 μm (upper panel), 2 μm (lower panel)

Next, the morphology of macrophages stimulated with various cytokines was observed by TEM (Fig. 5a). Unstimulated macrophages showed normal cell structure with a small circular nucleus. Similar cell morphology was observed after stimulation with IL-6/sIL-6R. By contrast, chromatin aggregation, nuclear fragmentation, plasma membrane blebbing, and cell shrinkage, consistent with apoptosis, were observed in actinomycin D-treated macrophages (Fig. 5a). Further, organelle and cell swelling was apparent after diamide treatment (upper panel in Fig. 5a), consistent with necroptosis, with a rupture of the cell membrane (lower panel in Fig. 5a). Organelles of macrophages treated with TNF-α were tumefied, similar to those treated with diamide (upper panel in Fig. 5a). The macrophage cell and intracellular organelles were enlarged, and further membrane rupture led to the release of the nucleus extracellularly and extracellular compartments intracellularly (lower panel in Fig. 5a). Nec-1, a necroptosis inhibitor, blocked the necroptotic effect of TNF-α (Additional file 2: Figure S2). TNF inhibitors ETN and ADA, and the JAK inhibitor TOF did not improve the cell morphology (organelle swelling) after diamide treatment of HD-derived macrophages (Additional file 2: Figure S3 and Figure S2) and RA-derived macrophages (data not shown, Additional file 2: Figure S4). These observations suggest that diamide and TNF-α both induced necroptosis in macrophages, via different pathways. Blocking TNF-α by the TNF inhibitor, but not JAK inhibitor, had a little effect on necroptosis triggered by TNF-α.

Next, we examined whether the signalling pathway involved in necroptosis was related to one of the key necroptosis proteins, receptor-interacting serine/threonine-protein kinase (RIP) 3. We analysed the phosphorylation of RIP3 (p-RIP3) in macrophages stimulated with TNF-α, diamide, and LPS/zVAD-FMK by ICC. No p-Akt (a control, serine/threonine-specific protein kinase) was detected in macrophages treated with TNF-α (Additional file 2: Figure S5). MLKL phosphorylation downstream of RIP3 (Fig. 5b) was next investigated by WB. Whereas no p-MLKL was detected in unstimulated macrophages, p-MLKL was observed from 2 to 24 h after TNF-α stimulation, with the p-MLKL signal reaching the maximum intensity after 8 h. Although the total amount of MLKL remained unchanged, MLKL phosphorylation was not detected in macrophages stimulated with IL-1β, IL-6/sIL-6R, or IL-21 (Additional file 2: Figure S6), or in cells treated with nec-1 prior to TNF-α stimulation (Additional file 2: Figure S7). In addition, some CD68 staining of the RA-derived synovial tissue was detected. While the membranes of some cells showed widespread and strong CD68 staining, others showed intermittent positive CD68 staining, indicating the possible cell death (data not shown).

**Fig. 6:**
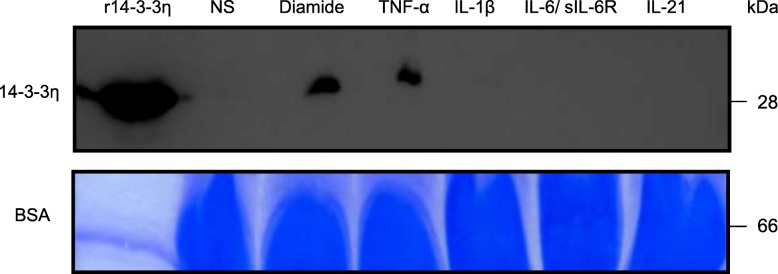
14-3-3η is detectable in culture supernatants of macrophages derived from HD and treated with TNF-α. The culture supernatants of macrophages cultured with or without diamide, TNF-α (100 ng/ml; *n* = 3), IL-1β (10 ng/ml; *n* = 3), IL-6/sIL-6R (10 ng/ml; *n* = 3), or IL-21 (10 ng/ml; *n* = 3) were subjected to WB. Recombinant 14-3-3η was used as a positive control. BSA was used as a loading control and was stained with CBB-R350. Representative images from three independent experiments are shown

At this stage of the investigation, it was unclear whether TNF-α-treated macrophages secreted 14-3-3η and wherein HD-derived macrophages stimulated with TNF-α 14-3-3η was localized. Therefore, we used WB to examine the levels of secreted 14-3-3η protein in concentrated macrophage culture supernatants (Fig. 6). 14-3-3η was not detected in the macrophage culture supernatant even when macrophages were treated with IL-1β, IL-6/sIL-6R, or IL-21, or pre-treated with nec-1 (Fig. 6 and Additional file 2: Figure S8). By contrast, a large amount of 14-3-3η was detected in the culture supernatant of HD-derived macrophages treated with diamide, TNF-α, or LPS/zVAD-FMK for 24 h.

## Discussion

In the current study, we examined the source of 14-3-3η in the RA synovial tissue by immunostaining and attempted to clarify the mechanism of macrophage cell death and 14-3-3η secretion into the inflamed synovia from patients with RA. We predicted two possible 14-3-3η secretion mechanisms: (1) exocytosis and (2) spontaneous secretion of the 14-3-3η protein after cell death. First, macrophages derived from HD monocytes were cultured in vitro. No 14-3-3η was detected in the culture supernatants in the presence or absence of various cytokine stimuli, such as IL-1β, IL-6/sIL-6R, and IL-21, except for TNF-α. Therefore, we concluded that the mechanism of 14-3-3η secretion is independent of exocytosis, since exocytosis does not cause secretion of 14-3-3η into the extracellular space. Next, we focused on TNF-α, which is important for the RA pathogenesis and elicits different types of cell death (apoptosis, necrosis, and necroptosis) depending on the cellular circumstances. TEM analysis revealed that resting macrophages or macrophages stimulated with IL-6/sIL-6R or TNF-α were not apoptotic. Furthermore, p-RIP3 and p-MLKL, markers of necroptosis, were detected in macrophages stimulated with TNF-α, indicating the role of TNF-α in macrophage necroptosis. Collectively, macrophages stimulated with TNF-α may suffer from necroptosis.

It has been reported that the level of glutathione is reduced in various cell types in patients with RA [33, 34]. We found that cell death induced in macrophages using diamide (which specifically oxidizes glutathione), LPS, or TNF-α was characterized by nuclear and organelle swelling, which was very similar to the events in cells undergoing necroptosis [35]. Based on the presented results, it appears that the cell death of macrophages in RA synovium occurs through necroptosis.

In addition, in a previous clinical study, we showed that the TNF-α inhibitor ADA strongly reduces the 14-3-3η levels in the serum of RA patients even before the patients achieve remission [21]. This suggested that the TNF-α pathway is closely related to the extracellular presence of 14-3-3η. To check the effect of TNF-α on 14-3-3η secretion, we here performed an ex vivo experiment using macrophages derived from HD monocytes. We observed that TNF-α stimulation induced necroptosis-like death of macrophages and 14-3-3η secretion into the culture supernatant. Taking together, we propose that in vivo, 14-3-3η is secreted by macrophages via necroptosis elicited by TNF-α.

It has been reported that a subset of genes, such as ones encoding TNF-α, IL-6, and MMP-3, is induced in the human monocytic cell line THP1 upon stimulation with recombinant 14-3-3η, resulting in inflammation [22]. The extracellular 14-3-3η may undergo citrullination, but the significance of this modification remains unknown. In a recent study, an immune complex consisting of extracellular citrullinated fibrinogen and ACPA was shown to activate macrophages by cross-linking with the TLR4 receptor and Fcγ on macrophages, which led to a strong induction of TNF-α [36]. Citrullination is an irreversible modification and is considered a crucial event that triggers the production of ACPA in RA. Although high 14-3-3η levels are observed in the RA synovial fluid, the mechanism of citrullinated 14-3-3η generation remains unknown. It has been reported that TNF-α activates PAD4 in the human oligodendroglial cell line, leading to the translocation of the latter to the nucleus [24]. We have previously shown that the serum levels of 14-3-3η are associated with the ACPA levels in patients with RA [21]. In the current study, we observed the co-localization of PAD4 and 14-3-3η in the nucleus of TNF-α-stimulated macrophages, indicating that 14-3-3η might be subjected to citrullination by TNF-α. 14-3-3 proteins play various roles in the cells, e.g., may be involved in gene expression and proteolysis. Hence, if the 14-3-3 protein undergoes citrullination, new target molecules of a possible citrullinated 14-3-3η could be proposed, which would attract the interest of researchers studying the 14-3-3 protein or ACPA production [16].

## Conclusions

The intracellular protein 14-3-3η is detected in the extracellular milieu in the synovial fluid of patients with RA. The goal of the current study was to identify the main source of 14-3-3η and the mechanism of its secretion into the extracellular space. Macrophages in the synovial tissue from RA, but not OA, exhibited widespread cytoplasmic staining for the 14-3-3η protein. Treatment with TNF-α, but not IL-6/sIL-6R, induced macrophage swelling and membrane rupture. This was followed by necroptosis. Further, high levels of 14-3-3η were detected in the culture supernatants of macrophages stimulated with TNF-α. These observations indicate that TNF-α promotes secretion of 14-3-3η by inducing necroptosis in macrophage, which constitutes a novel mechanism for 14-3-3η level increase in the RA synovial fluid.

## Supplementary information

**Additional file 1: Table S1.** Baseline demographics and clinical characteristics of patients with RA and OA. *ABT* abatacept*, ADA* adalimumab, *DAS28* Disease Activity Score based on 28-joints, *DMARD* isease modified anti-rheumatic-drugs*, ESR* erythrocyte sedimentation rate*, ETN* etanercept, *GC* Glucocorticosteroid, *IFX* infliximab, *MTX* methotrexate, *PSL* prednisolone, *mPSL* methylprednisolon, *N.A.* not applicable, *OA* osteoarthritis, *RA* rheumatoid arthritis, *SDAI* Simplified Disease Activity index, *TCZ* tocilizumab.

**Additional file 2: Figure S1.** Endogenous 14-3-3η levels in macrophages are not affected upon treatment with IL-1β, IL-6/sIL-6R, and IL-21. Macrophages were cultured in the presence or absence of IL-1β (10 ng/ml; *n* = 3), IL-6/sIL-6R (10 ng/ml; *n* = 3), or IL-21 (10 ng/ml; *n* = 3) for 24 h. WCL prepared from macrophages were then analysed by IB using specific antibodies against 14-3-3η or β-actin. **Figureure S2**. Nec-1, but not TOF, blocks TNF-α–induced macrophage death. Macrophages were cultured with TNF-α (100 ng/ml; *n* = 3) in the presence or absence of nec-1 (20 nM; n = 3) or TOF (300 nM; n = 3) for 24 h and analysed by TEM. Representative images from three independent experiments are shown. Scale bar, 5 μm (upper panel), 2 μm (lower panel). **Figure S3.** TNF inhibitors do not block macrophage death caused by diamide or TNF-α. Macrophages were cultured with diamide (1 mM; *n* = 3) or TNF-α (100 ng/ml; n = 3) in the presence or absence of ETN (100 μg/ml) or ADA (100 μg/ml) for 24 h and analysed by TEM. Representative images from three independent experiments are shown. Scale bar, 5 μm (upper panel), 2 μm (lower panel). **Figure S4.** TNF inhibitors do not block RA macrophage death caused by TNF-α. HD or RA macrophages were cultured in the presence or absence of TNF-α (100 ng/ml; *n* = 3) with or without ETN (100 μg/ml) or ADA (100 μg/ml) for 24 h and analysed by TEM. Representative images from three independent experiments are shown. Scale bar, 5 μm (upper panel), 2 μm (lower panel). **Figure S5.** TNF-α induces phosphorylation of RIP3. Macrophages were cultured with or without TNF-α (10 ng/ml; 24 h; n = 3), diamide (100 nM; 24h; n = 3) **(A)**, and LPS (500 ng/ml; 24 h; n = 3) in the presence or absence of zVAD-FMK (20 μM; n = 3). The cells were stained with specific antibodies against anti-RIP3 (phosphor S227) or phospho-Akt (Ser 473), or isotype control, and with DAPI. Representative images from three independent experiments are shown. Scale bar, 50 μm. **Figure S6.** IL-1β, IL-6/sIL-6R, and IL-21 fail to phosphorylate MLKL. Macrophages were cultured with or without IL-1β (10 ng/ml; n = 3), IL-6/sIL-6R (10 ng/ml; n = 3), or IL-21 (10 ng/ml; n = 3) for 24 h. WCL prepared from macrophages were analysed by IB using specific antibodies against the total or phosphorylated form of MLKL or β-actin. Quantification data for representative images from three independent experiments (n = 3) are shown. Scale bar, 5 μm (upper panel), 2 μm (lower panel). **Figure S7.** Nec-1 blocks phosphorylation of MLKL induced by TNF-α. Macrophages were cultured with or without TNF-α (10 ng/ml; 24 h; n = 3) in the presence or absence of nec-1 (20 nM; n = 3). WCL were then obtained, and total or phosphorylated form of MLKL, and β-actin were detected by WB. Representative images from three independent experiments are shown. **Figure S8.** 14-3-3η is detectable in culture supernatants of macrophages derived from HD and treated with TNF-α, diamide, or LPS. The culture supernatants of macrophages cultured in the presence or absence of diamide, TNF-α (10 ng/ml; 24 h; n = 3), or LPS (500 ng/ml; 24 h; n = 3), and with or without nec-1 (20 nM; n = 3) or TOF (300 nM; n = 3) or zVAD-FMK (20 μM; n = 3), were analysed by WB. Recombinant 14-3-3η was used as a positive control. BSA was used as a loading control and was stained with CBB-R350. Representative images from three independent experiments are shown.

## Data Availability

Not applicable

## Abbreviations

ABT: Abatacept
ACPA: Anti-cyclic citrullinated peptide antibody
ADA: Adalimumab
CBB: Coomassie Brilliant Blue
CDAI: Clinical Disease Activity Index
DAB: 3,3′,4,4′ Diaminobenzidine
DAPI: 4′,6-Diamidino-2-phenylindole
DAS28: Disease Activity Score of 28 joints
EDTA: Ethylenediaminetetraacetic acid
ESR: Erythrocyte sedimentation rate
ETN: Etanercept
FITC: Fluorescein isothiocyanate
GC: Glucocorticosteroid
HD: Healthy donor
HRP: Horseradish peroxidase
IB: Immunoblotting
ICC: Immunocytochemistry
IF: Immunofluorescence
IFX: Infliximab
IHC: Immunohistochemistry
IL: Interleukin
JAK: Janus kinase
LPS: Lipopolysaccharide
Lt: Left
MLKL: Mixed-lineage kinase domain-like pseudokinase
MMP: Matrix metalloproteinase
mPSL: Methylprednisolon
MTX: Methotrexate
N.a.: Not applicable
Nec-1: Necrostatin-1
OA: Osteoarthritis
PAD: Peptidylarginine deiminase
PBS: Phosphate-buffered saline
PBST: Phosphate-buffered saline containing 0.05% (v/v) Tween 20
PSL: Prednisolon
RA: Rheumatoid arthritis
RANKL: Receptor activator of nuclear factor kappa-Β ligand
RIP: Receptor-interacting serine/threonine-protein kinase
Rt: Right
SD: Standard deviation
SDAI: Simplified Disease Activity Index
sIL-6R: Soluble interleukin 6 receptor
STAT: Signal transducers and activators of transcription
TCZ: Tocilizumab
TEM: Transmission electron microscopy
TNF: Tumour necrosis factor
TOF: Tofacitinib
WB: Western blotting
WCL: Whole-cell lysate

## References

[1] McInnes IB, Schett G (2017). Pathogenetic insights from the treatment of rheumatoid arthritis. Lancet.

[2] Smolen JS, Aletaha D, McInnes IB (2016). Rheumatoid arthritis. Lancet.

[3] Schett G, Elewaut D, McInnes IB, Dayer JM, Neurath MF (2013). How cytokine networks fuel inflammation: toward a cytokine-based disease taxonomy. Nat Med.

[4] Tanaka Y (2012). Intensive treatment and treatment holiday of TNF-inhibitors in rheumatoid arthritis. Curr Opin Rheumatol.

[5] Tanaka Y (2019). Clinical immunity in bone and joints. J Bone Miner Metabolism.

[6] Redlich K, Smolen JS (2012). Inflammatory bone loss: pathogenesis and therapeutic intervention. Nat Rev Drug Discov.

[7] McInnes IB, Schett G (2011). The pathogenesis of rheumatoid arthritis. N Engl J Med.

[8] Kubo S, Yamaoka K, Kondo M, Yamagata K, Zhao J, Iwata S (2014). The JAK inhibitor, tofacitinib, reduces the T cell stimulatory capacity of human monocyte-derived dendritic cells. Ann Rheum Dis.

[9] Singh JA, Saag KG, Bridges SL, Akl EA, Bannuru RR, Sullivan MC (2016). American College of Rheumatology. 2015 American College of Rheumatology guideline for the treatment of rheumatoid arthritis. Arthritis Rheumatol.

[10] Smolen JS, Landewé R, Breedveld FC, Buch M, Burmester G, Dougados M (2014). EULAR recommendations for the management of rheumatoid arthritis with synthetic and biological disease-modifying antirheumatic drugs: 2013 update. Ann Rheum Dis.

[11] Tanaka Y (2019). The JAK inhibitors: do they bring a paradigm shift for the management of rheumatic diseases?. Rheumatology.

[12] Tanaka Y (2015). Recent progress and perspective in JAK inhibitors for rheumatoid arthritis: from bench to bedside. J Biochem.

[13] Kilani RT, Maksymowych WP, Aitken A, Boire G, St-Pierre Y, Li Y (2007). Detection of high levels of 2 specific isoforms of 14-3-3 proteins in synovial fluid from patients with joint inflammation. J Rheumatol.

[14] Fu H, Subramanian RR, Masters SC (2000). 14-3-3 proteins: structure, function, and regulation. Ann Rev Pharmacol Toxicol.

[15] Muslin AJ, Tanner JW, Allen PM, Shaw AS (1996). Interaction of 14-3-3 with signaling proteins is mediated by the recognition of phosphoserine. Cell.

[16] Jia H, Liang Z, Zhang X, Wang J, Xu W, Qian H (2017). 14-3-3 proteins: an important regulator of autophagy in diseases. Am J Transl Res.

[17] Cao W, Yang X, Zhou J, Teng Z, Cao L, Zhang X (2010). Targeting 14-3-3 protein, difopein induces apoptosis of human glioma cells and suppresses tumor growth in mice. Apoptosis.

[18] Maksymowych WP, Marotta A (2014). 14-3-3η: a novel biomarker platform for rheumatoid arthritis. Clin Exp Rheumatol.

[19] van Beers-Tas MH, Marotta A, Boers M, Maksymowych WP, van Schaardenburg D (2016). A prospective cohort study of 14-3-3η in ACPA and/or RF-positive patients with arthralgia. Arthritis Res Ther.

[20] Carrier N, Marotta A, de Brum-Fernandes AJ, Liang P, Masetto A, MénardHA (2016). Serum levels of 14-3-3η protein supplement C-reactive protein and rheumatoid arthritis-associated antibodies to predict clinical and radiographic outcomes in a prospective cohort of patients with recent-onset inflammatory polyarthritis. Arthritis Res Ther..

[21] Hirata S, Marotta A, Gui Y, Hanami K, Tanaka Y (2015). Serum 14-3-3η level is associated with severity and clinical outcomes of rheumatoid arthritis, and its pretreatment level is predictive of DAS28 remission with tocilizumab. Arthritis Res Ther..

[22] Maksymowych WP, van der Heijde D, Allaart CF, Landewé R, Boire G, Tak PP (2014). 14-3-3η is a novel mediator associated with the pathogenesis of rheumatoid arthritis and joint damage. Arthritis Res Ther..

[23] Mastronardi FG, Wood DD, Mei J, Raijmakers R, Tseveleki V, Dosch HM (2006). Increased citrullination of histone H3 in multiple sclerosis brain and animal models of demyelination: a role for tumor necrosis factor-induced peptidylarginine deiminase 4 translocation. J Neurosci.

[24] Lee SH, Kwon JY, Kim SY, Jung K, Cho ML (2017). Interferon-gamma regulates inflammatory cell death by targeting necroptosis in experimental autoimmune arthritis. Sci Rep.

[25] Miura M, Friedlander RM, Yuan J (1995). Tumor necrosis factor-induced apoptosis is mediated by a CrmA-sensitive cell death pathway. Proc Natl Acad Sci U S A.

[26] Vercammen D, Beyaert R, Denecker G, Goossens V, Van Loo G, Declercq W (1998). Inhibition of caspases increases the sensitivity of L929 cells to necrosis mediated by tumor necrosis factor. J Exp Med.

[27] Holler N, Zaru R, Micheau O, Thome M, Attinger A, Valitutti S (2000). Fas triggers an alternative, caspase-8-independent cell death pathway using the kinase RIP as effector molecule. Nat Immunol.

[28] Zhang J, Guo L, Liu M, Jing Y, Zhou S, Li H (2018). Receptor-interacting protein kinase 3 mediates macrophage/monocyte activation in autoimmune hepatitis and regulates interleukin-6 production. United European Gastroenterol J.

[29] Yamagata K, Li X, Ikegaki S, Oneyama C, Okada M, Nishita M (2012). Dissection of Wnt5a-Ror2 signaling leading to matrix metalloproteinase (MMP-13) expression. J Biol Chem.

[30] Li X, Yamagata K, Nishita M, Endo M, Arfian N, Rikitake Y (2013). Activation of Wnt5a-Ror2 signaling associated with epithelial-to-mesenchymaltransition of tubular epithelial cells during renal fibrosis. Genes Cells.

[31] Sonomoto K, Yamaoka K, Kaneko H, Yamagata K, Sakata K, Zhang X (2016). Spontaneous differentiation of human mesenchymal stem cells on poly-lactic-co-glycolic acid nano-fiber scaffold. PLoS One.

[32] Abramoff MD, Magelhaes PJ, Ram SJ (2004). Image processing with ImageJ. Biophoton Int.

[33] Chen R, Xu J, She Y, Jiang T, Zhou S, Shi H (2018). Necrostatin-1 protects C2C12 myotubes from CoCl2-induced hypoxia. Int J Mol Med.

[34] Damgaard D, Bjørn ME, Steffensen MA, Pruijn GJ, Nielsen CH (2016). Reduced glutathione as a physiological co-activator in the activation of peptidylarginine deiminase. Arthritis Res Ther..

[35] Dhuriya YK, Sharma D (2018). Necroptosis: a regulated inflammatory mode of cell death. J Neuroinflammation.

[36] Sokolove J, Zhao X, Chandra PE, Robinson WH (2011). Immune complexes containing citrullinated fibrinogen costimulate macrophages via Toll-like receptor 4 and Fcγ receptor. Arthritis Rheum.

