# Supplementary figures and images for: Tumour necrosis factor alpha promotes secretion of 14-3-3η by inducing necroptosis in macrophages
Source: Arthritis Res Ther. 2020 Feb 12;22:24. doi: 10.1186/s13075-020-2110-9 (PMC7017620; doi:10.1186/s13075-020-2110-9)

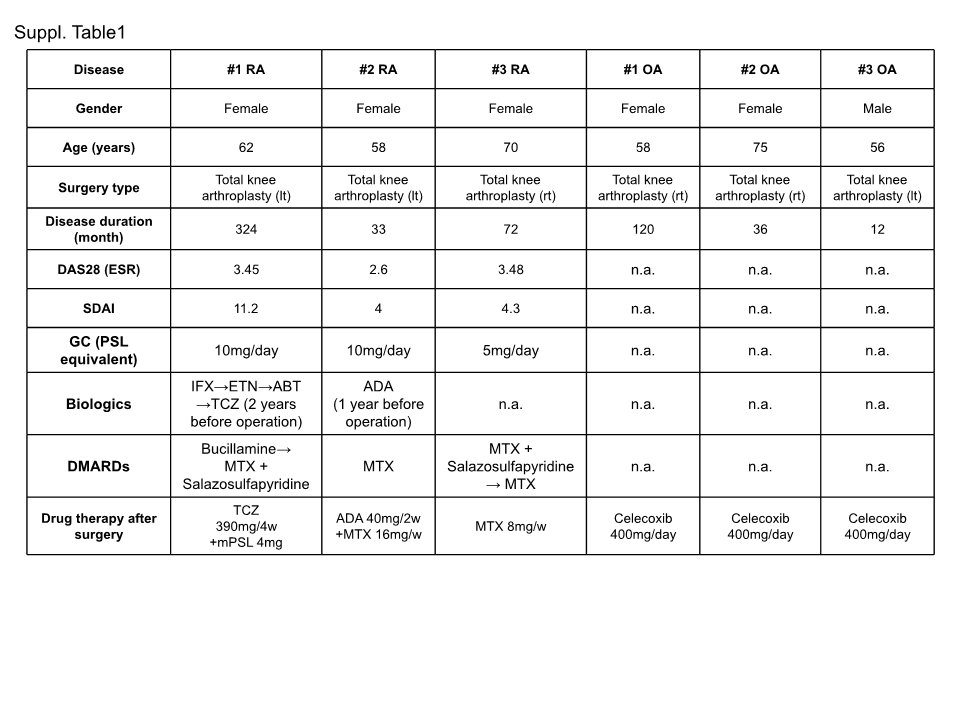

Supplement: Supplementary file 1 — Additional file 1: Table S1. Baseline demographics and clinical characteristics of patients with RA and OA. ABT abatacept, ADA adalimumab, DAS28 Disease Activity Score based on 28-joints, DMARD isease modified anti-rheumatic-drugs, ESR erythrocyte sedimentation rate, ETN etanercept, GC Glucocorticosteroid, IFX infliximab, MTX methotrexate, PSL prednisolone, mPSL methylprednisolon, N.A. not applicable, OA osteoarthritis, RA rheumatoid arthritis, SDAI Simplified Disease Activity index, TCZ tocilizumab. [file 13075_2020_2110_MOESM1_ESM.tiff]

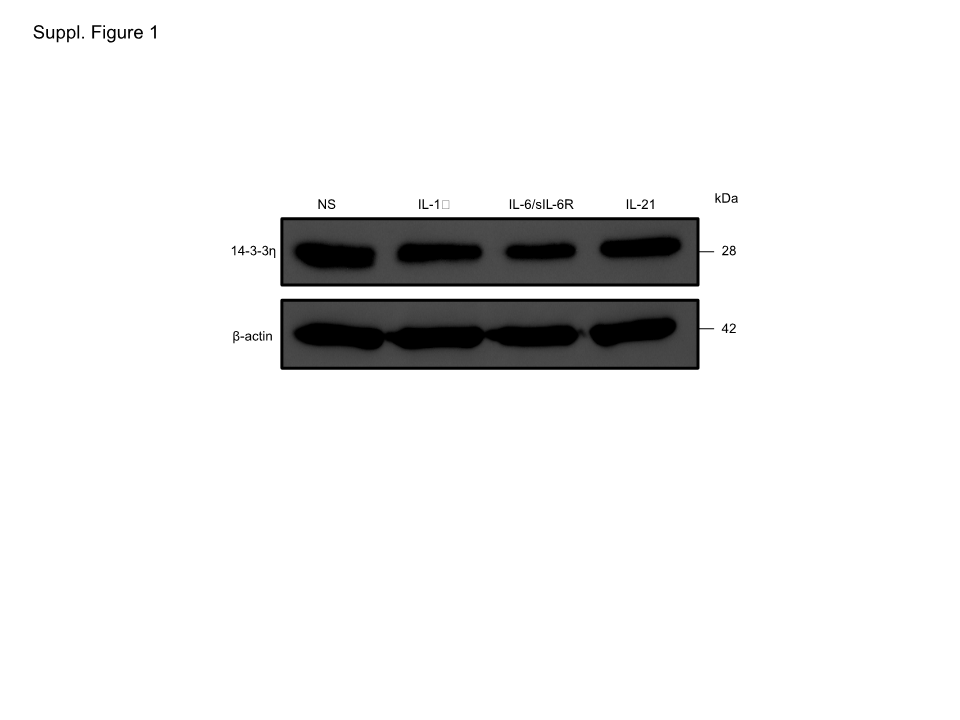

Supplement: Supplementary file 2 — Additional file 2: Figure S1. Endogenous 14-3-3η levels in macrophages are not affected upon treatment with IL-1β, IL-6/sIL-6R, and IL-21. Macrophages were cultured in the presence or absence of IL-1β (10 ng/ml; n = 3), IL-6/sIL-6R (10 ng/ml; n = 3), or IL-21 (10 ng/ml; n = 3) for 24 h. WCL prepared from macrophages were then analysed by IB using specific antibodies against 14-3-3η or β-actin. Figureure S2. Nec-1, but not TOF, blocks TNF-α–induced macrophage death. Macrophages were cultured with TNF-α (100 ng/ml; n = 3) in the presence or absence of nec-1 (20 nM; n = 3) or TOF (300 nM; n = 3) for 24 h and analysed by TEM. Representative images from three independent experiments are shown. Scale bar, 5 μm (upper panel), 2 μm (lower panel). Figure S3. TNF inhibitors do not block macrophage death caused by diamide or TNF-α. Macrophages were cultured with diamide (1 mM; n = 3) or TNF-α (100 ng/ml; n = 3) in the presence or absence of ETN (100 μg/ml) or ADA (100 μg/ml) for 24 h and analysed by TEM. Representative images from three independent experiments are shown. Scale bar, 5 μm (upper panel), 2 μm (lower panel). Figure S4. TNF inhibitors do not block RA macrophage death caused by TNF-α. HD or RA macrophages were cultured in the presence or absence of TNF-α (100 ng/ml; n = 3) with or without ETN (100 μg/ml) or ADA (100 μg/ml) for 24 h and analysed by TEM. Representative images from three independent experiments are shown. Scale bar, 5 μm (upper panel), 2 μm (lower panel). Figure S5. TNF-α induces phosphorylation of RIP3. Macrophages were cultured with or without TNF-α (10 ng/ml; 24 h; n = 3), diamide (100 nM; 24h; n = 3) (A), and LPS (500 ng/ml; 24 h; n = 3) in the presence or absence of zVAD-FMK (20 μM; n = 3). The cells were stained with specific antibodies against anti-RIP3 (phosphor S227) or phospho-Akt (Ser 473), or isotype control, and with DAPI. Representative images from three independent experiments are shown. Scale bar, 50 μm. Figure S6. IL-1β, IL-6/sIL-6R, a [file 13075_2020_2110_MOESM2_ESM.zip › Additional file 1.tiff]

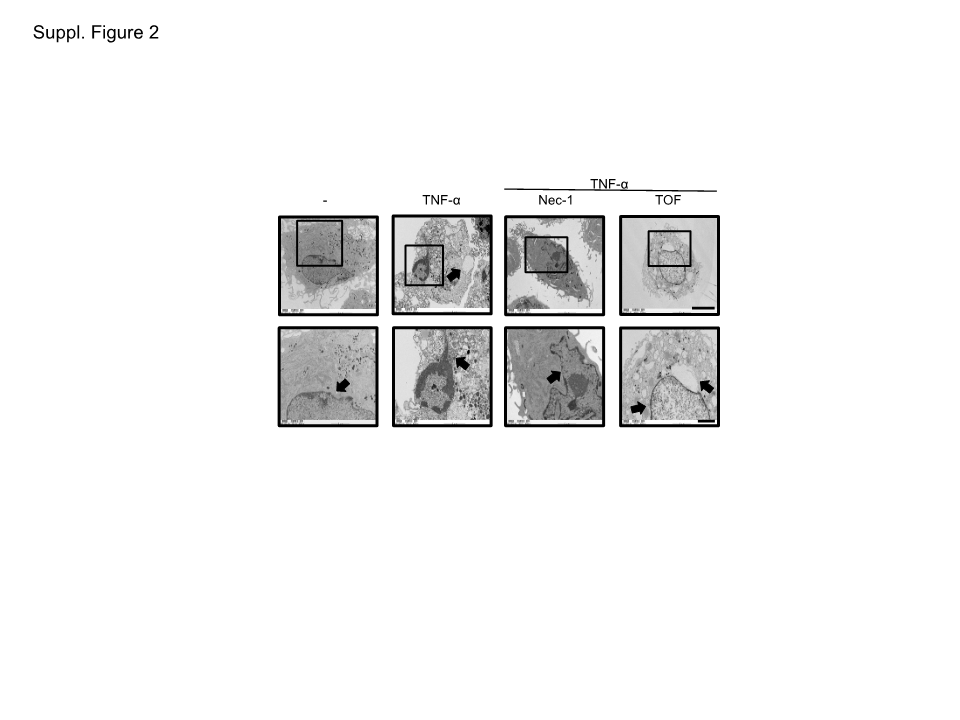

Supplement: Supplementary file 2 — Additional file 2: Figure S1. Endogenous 14-3-3η levels in macrophages are not affected upon treatment with IL-1β, IL-6/sIL-6R, and IL-21. Macrophages were cultured in the presence or absence of IL-1β (10 ng/ml; n = 3), IL-6/sIL-6R (10 ng/ml; n = 3), or IL-21 (10 ng/ml; n = 3) for 24 h. WCL prepared from macrophages were then analysed by IB using specific antibodies against 14-3-3η or β-actin. Figureure S2. Nec-1, but not TOF, blocks TNF-α–induced macrophage death. Macrophages were cultured with TNF-α (100 ng/ml; n = 3) in the presence or absence of nec-1 (20 nM; n = 3) or TOF (300 nM; n = 3) for 24 h and analysed by TEM. Representative images from three independent experiments are shown. Scale bar, 5 μm (upper panel), 2 μm (lower panel). Figure S3. TNF inhibitors do not block macrophage death caused by diamide or TNF-α. Macrophages were cultured with diamide (1 mM; n = 3) or TNF-α (100 ng/ml; n = 3) in the presence or absence of ETN (100 μg/ml) or ADA (100 μg/ml) for 24 h and analysed by TEM. Representative images from three independent experiments are shown. Scale bar, 5 μm (upper panel), 2 μm (lower panel). Figure S4. TNF inhibitors do not block RA macrophage death caused by TNF-α. HD or RA macrophages were cultured in the presence or absence of TNF-α (100 ng/ml; n = 3) with or without ETN (100 μg/ml) or ADA (100 μg/ml) for 24 h and analysed by TEM. Representative images from three independent experiments are shown. Scale bar, 5 μm (upper panel), 2 μm (lower panel). Figure S5. TNF-α induces phosphorylation of RIP3. Macrophages were cultured with or without TNF-α (10 ng/ml; 24 h; n = 3), diamide (100 nM; 24h; n = 3) (A), and LPS (500 ng/ml; 24 h; n = 3) in the presence or absence of zVAD-FMK (20 μM; n = 3). The cells were stained with specific antibodies against anti-RIP3 (phosphor S227) or phospho-Akt (Ser 473), or isotype control, and with DAPI. Representative images from three independent experiments are shown. Scale bar, 50 μm. Figure S6. IL-1β, IL-6/sIL-6R, a [file 13075_2020_2110_MOESM2_ESM.zip › Additional file 2.tiff]

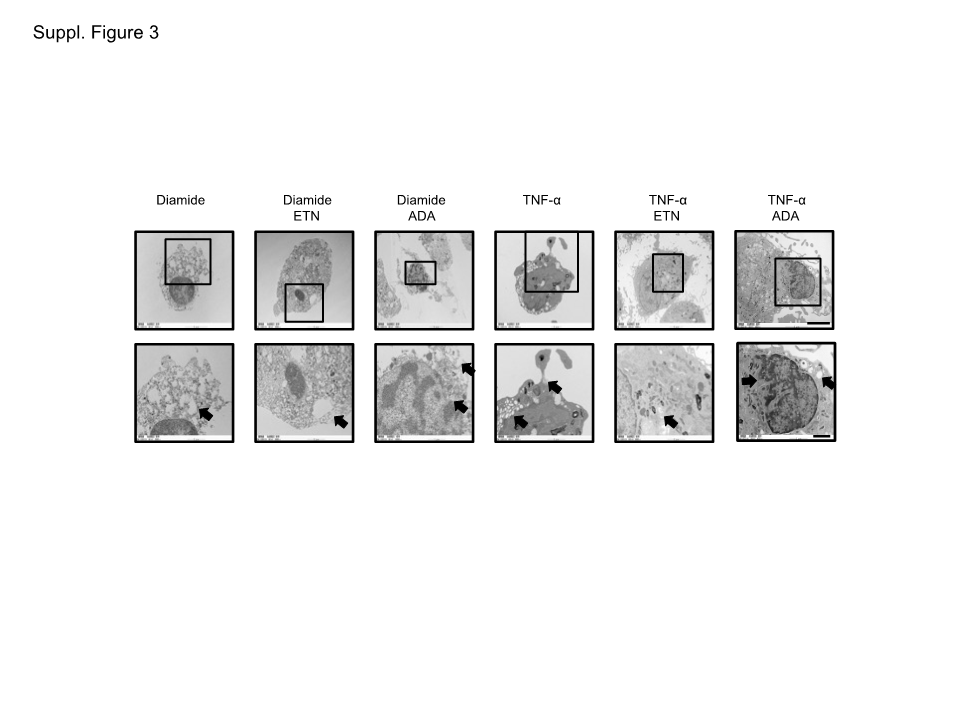

Supplement: Supplementary file 2 — Additional file 2: Figure S1. Endogenous 14-3-3η levels in macrophages are not affected upon treatment with IL-1β, IL-6/sIL-6R, and IL-21. Macrophages were cultured in the presence or absence of IL-1β (10 ng/ml; n = 3), IL-6/sIL-6R (10 ng/ml; n = 3), or IL-21 (10 ng/ml; n = 3) for 24 h. WCL prepared from macrophages were then analysed by IB using specific antibodies against 14-3-3η or β-actin. Figureure S2. Nec-1, but not TOF, blocks TNF-α–induced macrophage death. Macrophages were cultured with TNF-α (100 ng/ml; n = 3) in the presence or absence of nec-1 (20 nM; n = 3) or TOF (300 nM; n = 3) for 24 h and analysed by TEM. Representative images from three independent experiments are shown. Scale bar, 5 μm (upper panel), 2 μm (lower panel). Figure S3. TNF inhibitors do not block macrophage death caused by diamide or TNF-α. Macrophages were cultured with diamide (1 mM; n = 3) or TNF-α (100 ng/ml; n = 3) in the presence or absence of ETN (100 μg/ml) or ADA (100 μg/ml) for 24 h and analysed by TEM. Representative images from three independent experiments are shown. Scale bar, 5 μm (upper panel), 2 μm (lower panel). Figure S4. TNF inhibitors do not block RA macrophage death caused by TNF-α. HD or RA macrophages were cultured in the presence or absence of TNF-α (100 ng/ml; n = 3) with or without ETN (100 μg/ml) or ADA (100 μg/ml) for 24 h and analysed by TEM. Representative images from three independent experiments are shown. Scale bar, 5 μm (upper panel), 2 μm (lower panel). Figure S5. TNF-α induces phosphorylation of RIP3. Macrophages were cultured with or without TNF-α (10 ng/ml; 24 h; n = 3), diamide (100 nM; 24h; n = 3) (A), and LPS (500 ng/ml; 24 h; n = 3) in the presence or absence of zVAD-FMK (20 μM; n = 3). The cells were stained with specific antibodies against anti-RIP3 (phosphor S227) or phospho-Akt (Ser 473), or isotype control, and with DAPI. Representative images from three independent experiments are shown. Scale bar, 50 μm. Figure S6. IL-1β, IL-6/sIL-6R, a [file 13075_2020_2110_MOESM2_ESM.zip › Additional file 3.tiff]

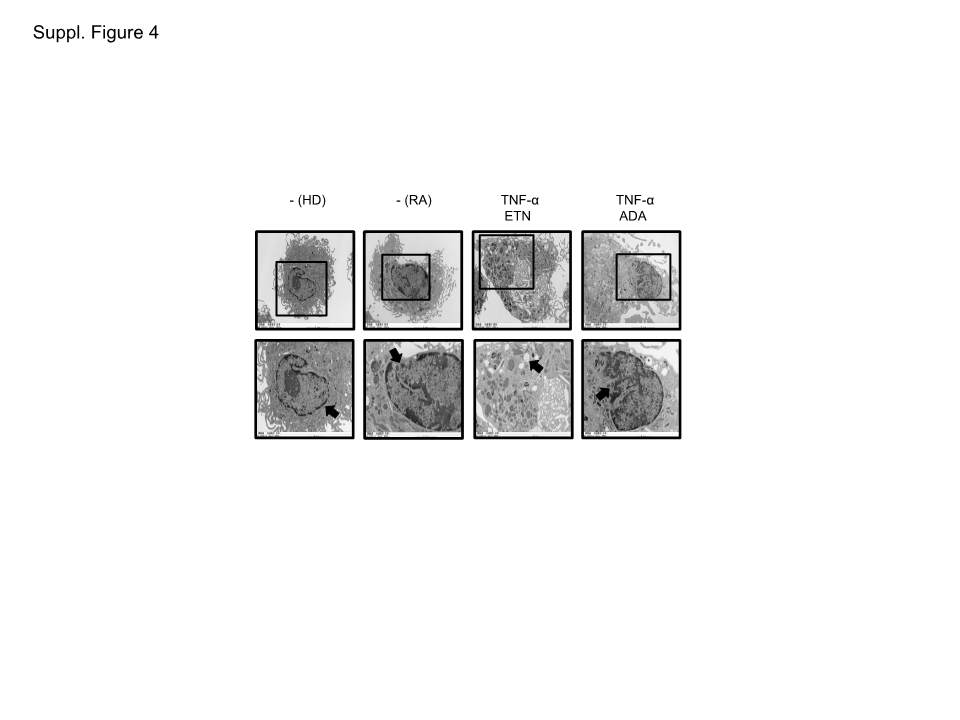

Supplement: Supplementary file 2 — Additional file 2: Figure S1. Endogenous 14-3-3η levels in macrophages are not affected upon treatment with IL-1β, IL-6/sIL-6R, and IL-21. Macrophages were cultured in the presence or absence of IL-1β (10 ng/ml; n = 3), IL-6/sIL-6R (10 ng/ml; n = 3), or IL-21 (10 ng/ml; n = 3) for 24 h. WCL prepared from macrophages were then analysed by IB using specific antibodies against 14-3-3η or β-actin. Figureure S2. Nec-1, but not TOF, blocks TNF-α–induced macrophage death. Macrophages were cultured with TNF-α (100 ng/ml; n = 3) in the presence or absence of nec-1 (20 nM; n = 3) or TOF (300 nM; n = 3) for 24 h and analysed by TEM. Representative images from three independent experiments are shown. Scale bar, 5 μm (upper panel), 2 μm (lower panel). Figure S3. TNF inhibitors do not block macrophage death caused by diamide or TNF-α. Macrophages were cultured with diamide (1 mM; n = 3) or TNF-α (100 ng/ml; n = 3) in the presence or absence of ETN (100 μg/ml) or ADA (100 μg/ml) for 24 h and analysed by TEM. Representative images from three independent experiments are shown. Scale bar, 5 μm (upper panel), 2 μm (lower panel). Figure S4. TNF inhibitors do not block RA macrophage death caused by TNF-α. HD or RA macrophages were cultured in the presence or absence of TNF-α (100 ng/ml; n = 3) with or without ETN (100 μg/ml) or ADA (100 μg/ml) for 24 h and analysed by TEM. Representative images from three independent experiments are shown. Scale bar, 5 μm (upper panel), 2 μm (lower panel). Figure S5. TNF-α induces phosphorylation of RIP3. Macrophages were cultured with or without TNF-α (10 ng/ml; 24 h; n = 3), diamide (100 nM; 24h; n = 3) (A), and LPS (500 ng/ml; 24 h; n = 3) in the presence or absence of zVAD-FMK (20 μM; n = 3). The cells were stained with specific antibodies against anti-RIP3 (phosphor S227) or phospho-Akt (Ser 473), or isotype control, and with DAPI. Representative images from three independent experiments are shown. Scale bar, 50 μm. Figure S6. IL-1β, IL-6/sIL-6R, a [file 13075_2020_2110_MOESM2_ESM.zip › Additional file 4.tiff]

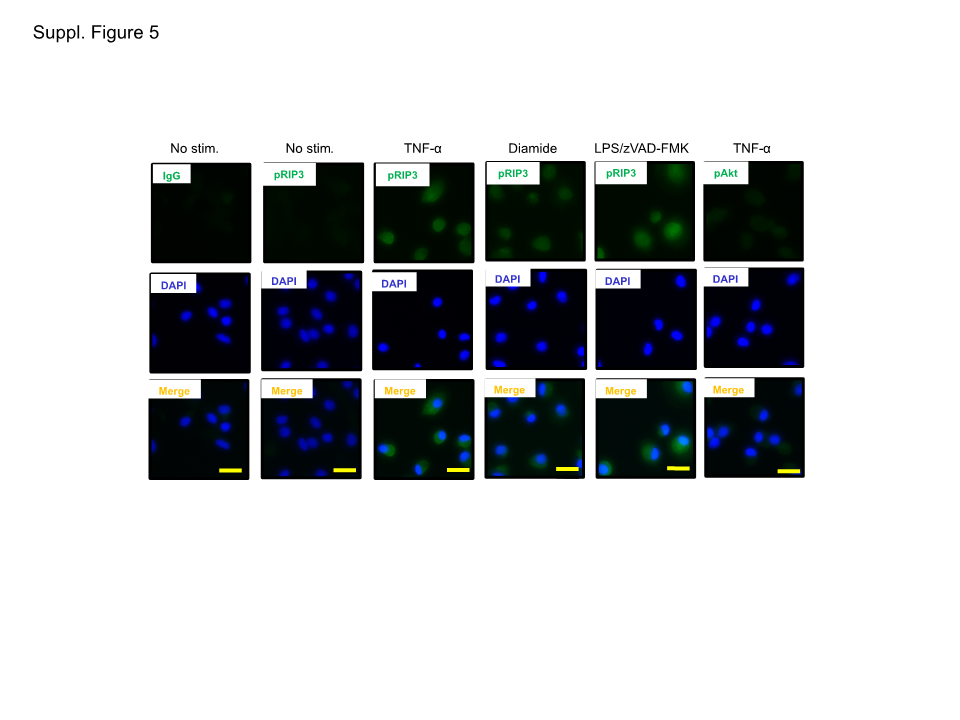

Supplement: Supplementary file 2 — Additional file 2: Figure S1. Endogenous 14-3-3η levels in macrophages are not affected upon treatment with IL-1β, IL-6/sIL-6R, and IL-21. Macrophages were cultured in the presence or absence of IL-1β (10 ng/ml; n = 3), IL-6/sIL-6R (10 ng/ml; n = 3), or IL-21 (10 ng/ml; n = 3) for 24 h. WCL prepared from macrophages were then analysed by IB using specific antibodies against 14-3-3η or β-actin. Figureure S2. Nec-1, but not TOF, blocks TNF-α–induced macrophage death. Macrophages were cultured with TNF-α (100 ng/ml; n = 3) in the presence or absence of nec-1 (20 nM; n = 3) or TOF (300 nM; n = 3) for 24 h and analysed by TEM. Representative images from three independent experiments are shown. Scale bar, 5 μm (upper panel), 2 μm (lower panel). Figure S3. TNF inhibitors do not block macrophage death caused by diamide or TNF-α. Macrophages were cultured with diamide (1 mM; n = 3) or TNF-α (100 ng/ml; n = 3) in the presence or absence of ETN (100 μg/ml) or ADA (100 μg/ml) for 24 h and analysed by TEM. Representative images from three independent experiments are shown. Scale bar, 5 μm (upper panel), 2 μm (lower panel). Figure S4. TNF inhibitors do not block RA macrophage death caused by TNF-α. HD or RA macrophages were cultured in the presence or absence of TNF-α (100 ng/ml; n = 3) with or without ETN (100 μg/ml) or ADA (100 μg/ml) for 24 h and analysed by TEM. Representative images from three independent experiments are shown. Scale bar, 5 μm (upper panel), 2 μm (lower panel). Figure S5. TNF-α induces phosphorylation of RIP3. Macrophages were cultured with or without TNF-α (10 ng/ml; 24 h; n = 3), diamide (100 nM; 24h; n = 3) (A), and LPS (500 ng/ml; 24 h; n = 3) in the presence or absence of zVAD-FMK (20 μM; n = 3). The cells were stained with specific antibodies against anti-RIP3 (phosphor S227) or phospho-Akt (Ser 473), or isotype control, and with DAPI. Representative images from three independent experiments are shown. Scale bar, 50 μm. Figure S6. IL-1β, IL-6/sIL-6R, a [file 13075_2020_2110_MOESM2_ESM.zip › Additional file 5.tiff]

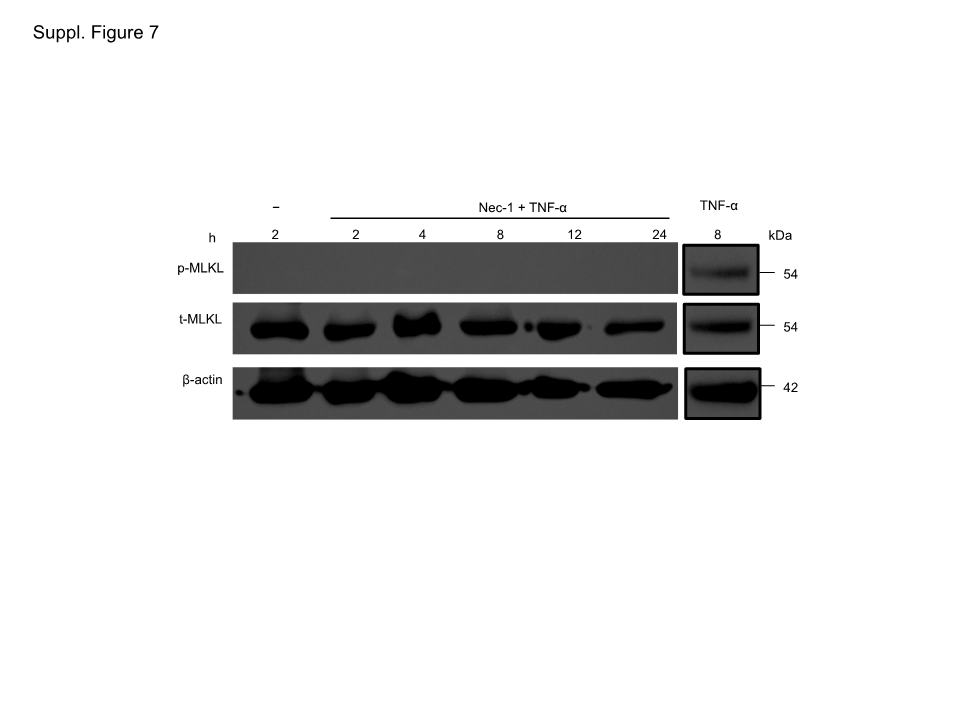

Supplement: Supplementary file 2 — Additional file 2: Figure S1. Endogenous 14-3-3η levels in macrophages are not affected upon treatment with IL-1β, IL-6/sIL-6R, and IL-21. Macrophages were cultured in the presence or absence of IL-1β (10 ng/ml; n = 3), IL-6/sIL-6R (10 ng/ml; n = 3), or IL-21 (10 ng/ml; n = 3) for 24 h. WCL prepared from macrophages were then analysed by IB using specific antibodies against 14-3-3η or β-actin. Figureure S2. Nec-1, but not TOF, blocks TNF-α–induced macrophage death. Macrophages were cultured with TNF-α (100 ng/ml; n = 3) in the presence or absence of nec-1 (20 nM; n = 3) or TOF (300 nM; n = 3) for 24 h and analysed by TEM. Representative images from three independent experiments are shown. Scale bar, 5 μm (upper panel), 2 μm (lower panel). Figure S3. TNF inhibitors do not block macrophage death caused by diamide or TNF-α. Macrophages were cultured with diamide (1 mM; n = 3) or TNF-α (100 ng/ml; n = 3) in the presence or absence of ETN (100 μg/ml) or ADA (100 μg/ml) for 24 h and analysed by TEM. Representative images from three independent experiments are shown. Scale bar, 5 μm (upper panel), 2 μm (lower panel). Figure S4. TNF inhibitors do not block RA macrophage death caused by TNF-α. HD or RA macrophages were cultured in the presence or absence of TNF-α (100 ng/ml; n = 3) with or without ETN (100 μg/ml) or ADA (100 μg/ml) for 24 h and analysed by TEM. Representative images from three independent experiments are shown. Scale bar, 5 μm (upper panel), 2 μm (lower panel). Figure S5. TNF-α induces phosphorylation of RIP3. Macrophages were cultured with or without TNF-α (10 ng/ml; 24 h; n = 3), diamide (100 nM; 24h; n = 3) (A), and LPS (500 ng/ml; 24 h; n = 3) in the presence or absence of zVAD-FMK (20 μM; n = 3). The cells were stained with specific antibodies against anti-RIP3 (phosphor S227) or phospho-Akt (Ser 473), or isotype control, and with DAPI. Representative images from three independent experiments are shown. Scale bar, 50 μm. Figure S6. IL-1β, IL-6/sIL-6R, a [file 13075_2020_2110_MOESM2_ESM.zip › Additional file 7.tiff]

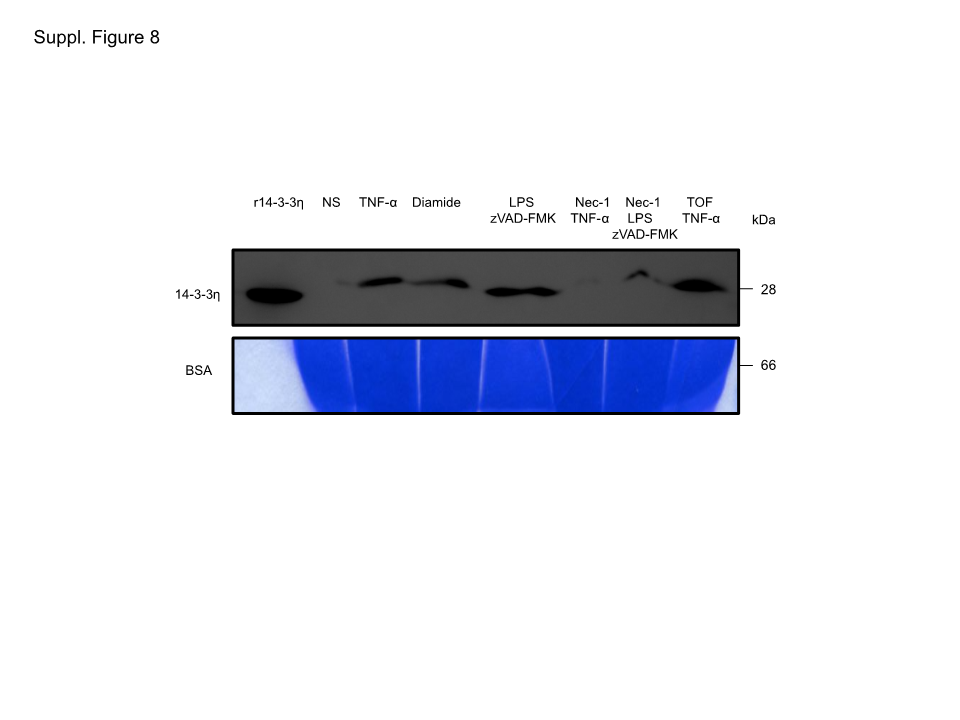

Supplement: Supplementary file 2 — Additional file 2: Figure S1. Endogenous 14-3-3η levels in macrophages are not affected upon treatment with IL-1β, IL-6/sIL-6R, and IL-21. Macrophages were cultured in the presence or absence of IL-1β (10 ng/ml; n = 3), IL-6/sIL-6R (10 ng/ml; n = 3), or IL-21 (10 ng/ml; n = 3) for 24 h. WCL prepared from macrophages were then analysed by IB using specific antibodies against 14-3-3η or β-actin. Figureure S2. Nec-1, but not TOF, blocks TNF-α–induced macrophage death. Macrophages were cultured with TNF-α (100 ng/ml; n = 3) in the presence or absence of nec-1 (20 nM; n = 3) or TOF (300 nM; n = 3) for 24 h and analysed by TEM. Representative images from three independent experiments are shown. Scale bar, 5 μm (upper panel), 2 μm (lower panel). Figure S3. TNF inhibitors do not block macrophage death caused by diamide or TNF-α. Macrophages were cultured with diamide (1 mM; n = 3) or TNF-α (100 ng/ml; n = 3) in the presence or absence of ETN (100 μg/ml) or ADA (100 μg/ml) for 24 h and analysed by TEM. Representative images from three independent experiments are shown. Scale bar, 5 μm (upper panel), 2 μm (lower panel). Figure S4. TNF inhibitors do not block RA macrophage death caused by TNF-α. HD or RA macrophages were cultured in the presence or absence of TNF-α (100 ng/ml; n = 3) with or without ETN (100 μg/ml) or ADA (100 μg/ml) for 24 h and analysed by TEM. Representative images from three independent experiments are shown. Scale bar, 5 μm (upper panel), 2 μm (lower panel). Figure S5. TNF-α induces phosphorylation of RIP3. Macrophages were cultured with or without TNF-α (10 ng/ml; 24 h; n = 3), diamide (100 nM; 24h; n = 3) (A), and LPS (500 ng/ml; 24 h; n = 3) in the presence or absence of zVAD-FMK (20 μM; n = 3). The cells were stained with specific antibodies against anti-RIP3 (phosphor S227) or phospho-Akt (Ser 473), or isotype control, and with DAPI. Representative images from three independent experiments are shown. Scale bar, 50 μm. Figure S6. IL-1β, IL-6/sIL-6R, a [file 13075_2020_2110_MOESM2_ESM.zip › Additional file 8.tiff]
